# Evaluation of probiotic characteristics and whole genome analysis of *Pediococcus pentosaceus* MR001 for use as probiotic bacteria in shrimp aquaculture

**DOI:** 10.1038/s41598-021-96780-z

**Published:** 2021-09-15

**Authors:** Warapond Wanna, Komwit Surachat, Panmile Kaitimonchai, Amornrat Phongdara

**Affiliations:** 1 Division of Biological Science, Faculty of Science, Prince of Songkla University, Hat Yai, Songkhla, 90110 Thailand; 2 Center for Genomics and Bioinformatics Research, Faculty of Science, Prince of Songkla University, Songkhla, 90110 Thailand; 3 Faculty of Medical Technology, Prince of Songkla University, Songkhla, 90110 Thailand; 4 Division of Computational Science, Faculty of Science, Prince of Songkla University, Songkhla, 90110 Thailand; 5 Molecular Evolution and Computational Biology Research Unit, Faculty of Science, Prince of Songkla University, Hat Yai, 90110 Songkhla Thailand

**Keywords:** Biotechnology, Molecular biology

## Abstract

The development of non-antibiotic and environmentally friendly agents is a key consideration for health management in shrimp aquaculture. In this study, the probiotic potential in shrimp aquaculture of *Pediococcus pentosaceus* MR001, isolated from *Macrobrachium rosenbergii*, was investigated by means of feeding trial and genetic characterization. In the feeding trial, dietary supplementation with *P. pentosaceus* MR001 significantly increased weight gain and digestive enzyme activity (*p* < 0.05) in shrimp, *Litopenaeus vannamei*. The intestinal histology showed that shrimp given the probiotic diet had healthier guts than the control group. Also, the immune gene expression and the survival rate in the treatment group were significantly increased when compared with the control group. The genetic characteristics of *P. pentosaceus* strain MR001 were explored by performing whole-genome sequencing (WGS) using the HiSeq 2500 platform and PacBio system, revealing the complete circular genome of 1,804,896 bp. We also identified 1789 coding genes and subsequently characterized genes related to the biosynthesis of bacteriocins, stress resistance, and bile tolerance. Our findings suggest that insights in the functional and genetic characteristics of *P. pentosaceus* strain MR001 could provide opportunities for applications of such strain in shrimp diet supplementation.

## Introduction

Epidemics are increasingly recognized in many countries as one of the most important constraints on cultivated shrimp production. The development of non-antibiotic and environmentally friendly agents is a key factor for health management in aquaculture^1^. To reduce the use of antibiotics while maintaining or even increasing production efficiency, many farmers feed probiotics in place of antibiotics to their aquatic animals. Probiotics are live microorganisms that play an important role in the microbial balance of the host organisms. They can reduce pathogenic microbes, stimulate growth rate, and improve animal health^1^. Nevertheless, it is essential that the effects of probiotics are thoroughly investigated prior to any aquaculture applications. A variety of effects are dependent on the probiotic itself, the dosage employed, the treatment duration and the route and frequency of delivery^2^. The most common probiotics used in aquaculture include lactic acid bacteria (LAB) such as *Lactobacillus* sp., *Bacillus* sp., *Pediococcus* sp., *Enterococcus* sp., and yeast (*Saccharomyces cerevisiae*)^3–6^. *Pediococcus pentosaceus* is often used in starter cultures for fermenting meat and vegetable products in the food industry^7,8^. Additionally, studies have shown that *P. pentosaceus* can enhance innate immunity, physiological health and resistance to pathogens in fish^4^ and shrimp^9,10^. Despite these findings, little is known about its genetic information and functions, compared with another closely related species *P. acidilactici*, which is widely used in aquaculture^11,12^.

Some complete genome sequences of *P. pentosaceus* have been made available, but the strains investigated were isolated from food and the human intestine. Hence, this study focuses on the strain of *P. pentosaceus* MR001, isolated from *Macrobrachium rosenbergii*, which showed probiotic properties in vitro. The dietary supplementation was evaluated, and the whole genome was characterized in order to further evaluate the potential of this strain as a probiotic dietary supplement in marine aquaculture.

## Results

### Isolation and characterization of *P. pentosaceus MR001*

Twelve LAB were isolated from giant freshwater prawn gut and showed antagonistic ability against *V. harveyi.* Two isolates (no.4 and no.8) that have the best inhibition and high hydrophobicity (> 80%) were identified (Supporting Fig. 1, Supporting Table 1). Molecular analysis of the isolates was tested using 16sRNA which showed that two isolates were identified as the *Pediococcus pentosaceus*. Isolate no. 4 was defined as MR001 and retained for further analysis.

**Figure 1:**
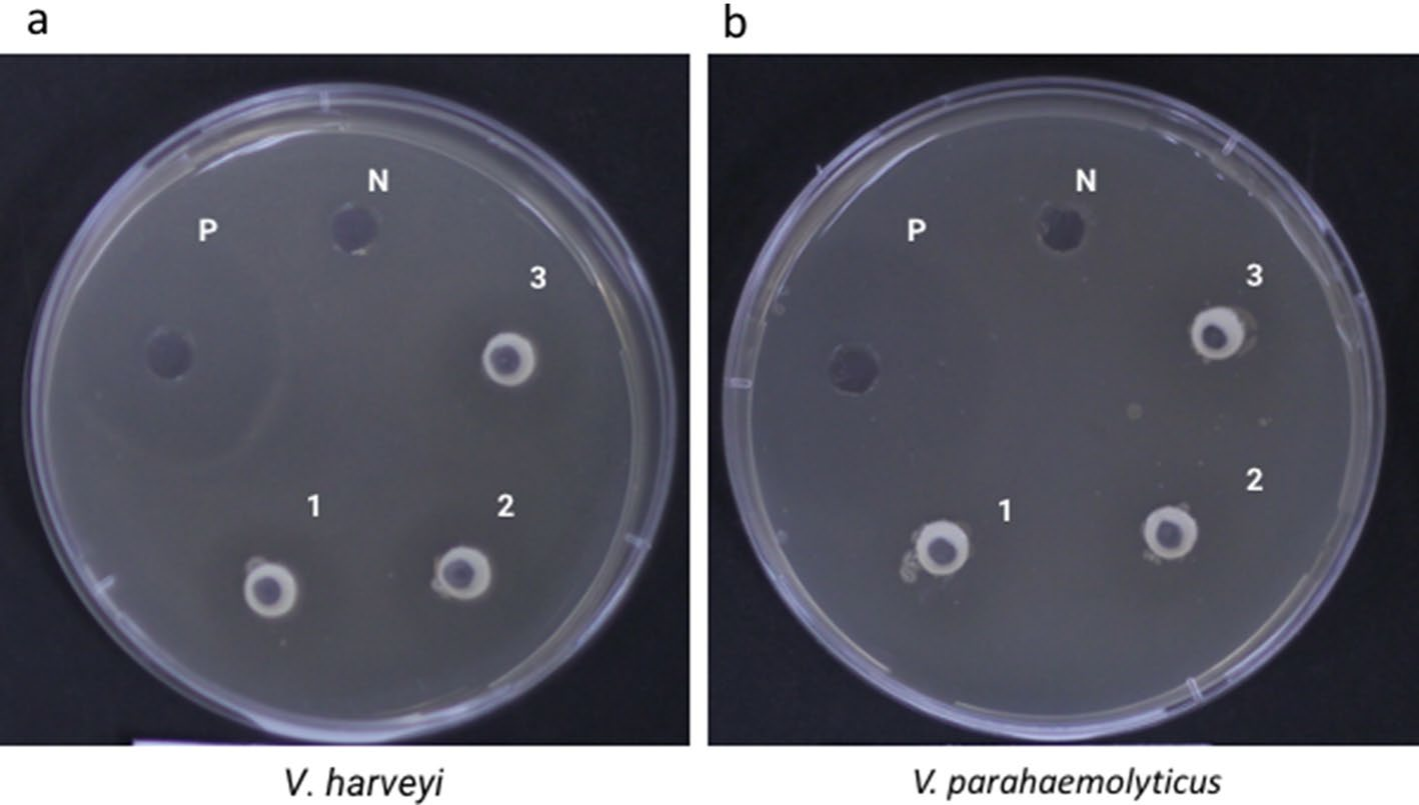
Inhibition zones produced by antimicrobial activity of *P. pentosaceus* MR001 on (**a**) *V. harveyi* and (**b**) *V. parahaemolyticus* were shown. P: positive control (100 μg/ml tetracycline), N: negative control (MRS broth), 1–3: culture supernatant.

*P. pentosaceus* MR001 is Gram-positive, and coccus-shaped. The MR001 recorded a viable count, 4–6 log10 CFU/mL at pH 3 (Table 1). It tolerates up to 6% NaCl in MRS broth (7.86 ± 0.09 log10 CFU/ml) (Table 2). One important property to be called MR001 as a probiotic is the ability to tolerate ingredients in the gastrointestinal tract like bile salt. For this experiment different concentrations of bile salt in MRS broth (0.6–1%) were prepared. The results showed that *P. pentosaceus* can tolerate bile salt in all those concentrations, up to 1% bile salt as shown in Table 2. MR001 also exhibited antibacterial activity against shrimp pathogens including *V. harveyi* and *V. parahaemolyticus* (Fig. 1).

**Table 1:** Total plate counts for *P. pentosaceus* MR001 on MRS agar at pH 3 and 7 (control) over 3 h intervals. Values are mean ± standard deviation (n = 3).

	Total plate count (log_10_ CFU/ml)		
	0 h	3 h	6 h
pH 3	6.73 ± 0.34	6.62 ± 0.41	4.98 ± 1.52
pH 7	7.35 ± 0.06	7.31 ± 0.13	8.08 ± 0.21

**Table 2:** Effect of NaCl and bile salt on the growth of *P. pentosaceus* MR001. Values are mean ± standard deviation (n = 3).

		Total plate count (log_10_ CFU/ml)
NaCl	1%	9.23 ± 0.04
	2%	9.58 ± 0.03
	3%	8.99 ± 0.13
	4%	8.89 ± 0.10
	5%	8.68 ± 0.21
	6%	7.86 ± 0.09
Bile salt	0.6%	7.52 ± 0.13
	0.8%	6.20 ± 0.03
	1%	5.87 ± 0.06

The auto-aggregation and co-aggregation properties of MR001 are shown in Table 3. The probiotic strains showed auto-aggregation values, ranging between 40 and 75% at 2–24 h incubation time. In addition, MR001 showed the high co-aggregation abilities with *V. harveyi* (70%) and *V. parahaemolyticus* (83%) at 24 h.

**Table 3:** Auto-aggregation and co-aggregation abilities of *P. pentosaceus* MR001. Values are mean ± standard deviation (n = 3).

	% of auto-aggregation		
	2 h	6 h	24 h
MR001	40.40 ± 1.07	72.03 ± 0.48	75.00 ± 0.97

	% of co-aggregation		
	2 h	6 h	24 h
*V. parahaemolyticus*	12.74 ± 0.21	50.81 ± 0.04	83.04 ± 0.13
*V. harveyi*	20.11 ± 0.13	53.47 ± 0.06	70.71 ± 0.08

### Growth performance of *L. vannamei*

Post-larvae of *L. vannamei* were divided into four groups. One group was given a control diet, and the rest were given diets containing the probiotic supplement at different concentrations. The results of growth performance and feed utilization are presented in Table 4. Shrimp treated with the probiotic bacteria at 10^9^ CFU/g exhibited significantly higher degrees of weight gain, a faster specific growth rate, and lower feed conversion ratio (FCR), compared with the control group (*p* < 0.05). However, no significant differences were observed between the groups that received probiotic supplementation at 10^7^ CFU/g and at 10^8^ CFU/g. Therefore, the group with probiotic supplementation at 10^9^ CFU/g was selected to further analyze the potential probiotic activity of *P. pentosaceus* MR001 in later experiments.

**Table 4:** Growth performance and feed utilization of *P. pentosaceus* MR001-treated *L. vannamei*. Different letters indicate significant comparison (*p* < 0.05).

	Control	Probiotic 10^7^	Probiotic 10^8^	Probiotic 10^9^
Initial weight (g)	4.106 ± 0.70	4.59 ± 0.36	4.31 ± 0.32	3.59 ± 0.21
Final weight (g)	4.73 ± 0.81	5.21 ± 0.41	5.06 ± 0.39	4.3 ± 0.19
Weight gain	13.66 ± 1.89^a^	13.29 ± 1.99^a^	15.53 ± 0.56^ab^	17.56 ± 1.40^b^
Feed efficiency	1.30 ± 0.18^a^	1.27 ± 0.19^a^	1.48 ± 0.05^ab^	1.67 ± 0.13^b^
FCR	0.79 ± 0.12^ab^	0.81 ± 0.11^a^	0.68 ± 0.02^ab^	0.60 ± 0.05^b^
SGR	0.61 ± 0.08^a^	0.59 ± 0.08^a^	0.69 ± 0.02^ab^	0.77 ± 0.06^b^

### Digestive enzyme activity

The digestive enzyme activity of *L. vannamei* was evaluated after a three-week feeding trial. The activities of trypsin, amylase and lipase were significantly enhanced in the 10^9^ CFU/g probiotic group, compared to the control group (*p* < 0.05). However, no differences were detected in chymotrypsin and cellulase activities (Table 5).

**Table 5:** Activity of digestive enzymes in the GIT of *L. vannamei* after the feeding experiment. Asterisks show a significant difference between groups (*p* < 0.05).

Digestive enzymes	Control	Probiotic
Trypsin (U mg protein^−1^)	0.89 ± 0.18	1.66 ± 0.46*
Chymotrypsin (mU mg protein^−1^)	10.94 ± 2.91	13.84 ± 5.29
Activity ratio of trypsin to chymotrypsin (T/C ratio)	69.70 ± 19.68	148.26 ± 54.71
Amylase (U mg protein^−1^)	780.09 ± 94.37	1660.92 ± 435.10*
Cellulase (U mg protein^−1^)	13.37 ± 1.75	15.71 ± 2.15
Lipase (U mg protein^–1^)	392.20 ± 118.68	1117.16 ± 172.33*
Total protein	11.04 ± 1.30	18.98 ± 4.39*

### Intestinal histology

**Figure 2:**
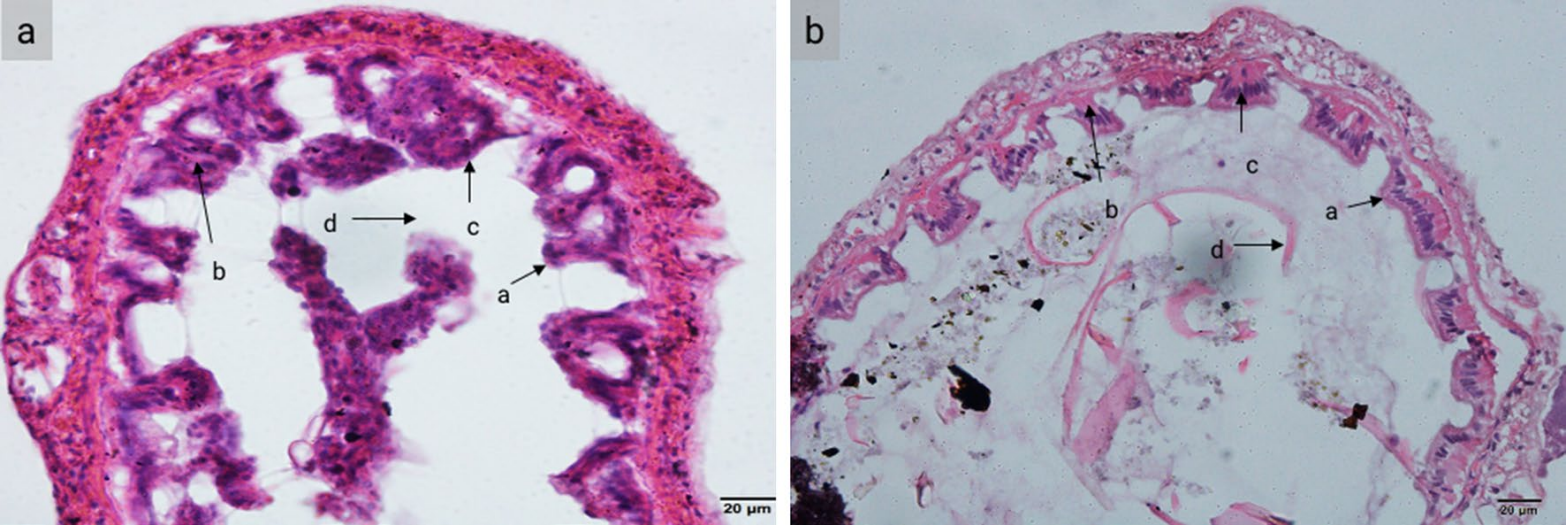
Histological sections of shrimp intestinal tissue (× 400). Shrimp had received (**a**) a diet supplemented with *P. pentosaceus* MR001 and (**b**) a basal diet. Abbreviations: a, brush border; b, epithelium; c, nuclei and d, lumen.

At the end of the three-week feeding trial, the intestinal tissue of shrimp that received probiotic dietary supplementation at 10^9^ CFU/g was collected and stained with hematoxylin and eosin (H&E). Compared with the commercial feed group, the tissue of the probiotic group showed more closely connected and longer epithelium (Fig. 2, Table 6).

**Table 6:** Intestinal histology of shrimp fed a diet supplement with the MR001 probiotic for 3 weeks. Asterisks show a significant difference between groups (*p* < 0.05).

Muscular layer	Diets	
	No Probiotic	Probiotic
Thickness (µm)	25.18 ± 2.04	42.55 ± 7.46*
Villi height (µm)	21.48 ± 1.81	49.12 ± 6.81*

### Cumulative survival rate of *L. vannamei* after *V. parahaemolyticus* infection

**Figure 3:**
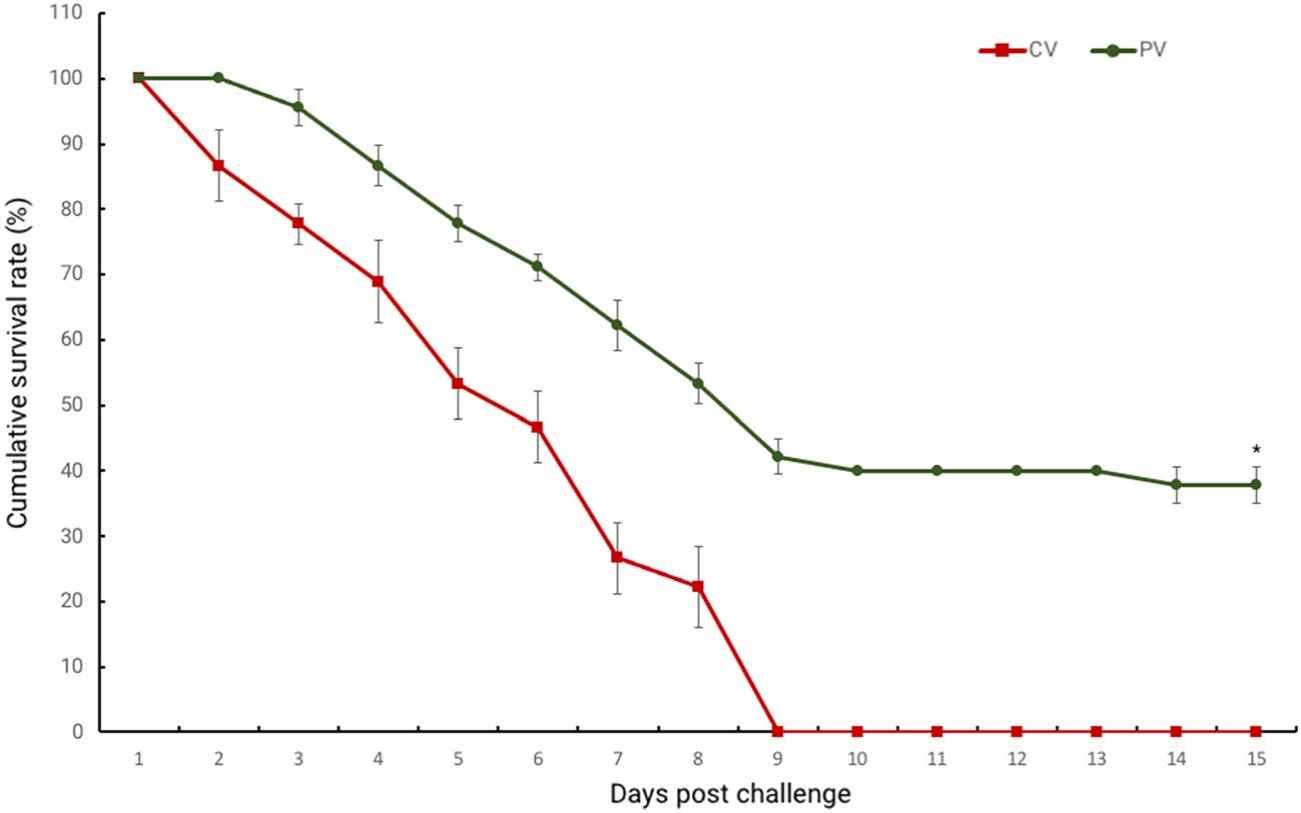
The survival rate of *P. pentosaceus* MR001-treated *L. vannamei* challenged with *V. parahaemolyticus*. Error bars indicate standard deviations (n = 3). Asterisks indicate significant difference comparisons (*p* < 0.05).

The survival rate of the shrimp receiving the probiotic bacteria at 10^9^ CFU/g was determined after an infection challenge with *V. parahaemolyticus* (PV group). The PV group showed a higher survival rate than the infection-challenged group that received the commercial diet without probiotic (CV group), with the difference being 40% on day 10 post-infection. After 10 days-post challenge, the percent of survival rate reached a plateau (Fig. 3).

### Immune-related gene expression

**Figure 4:**
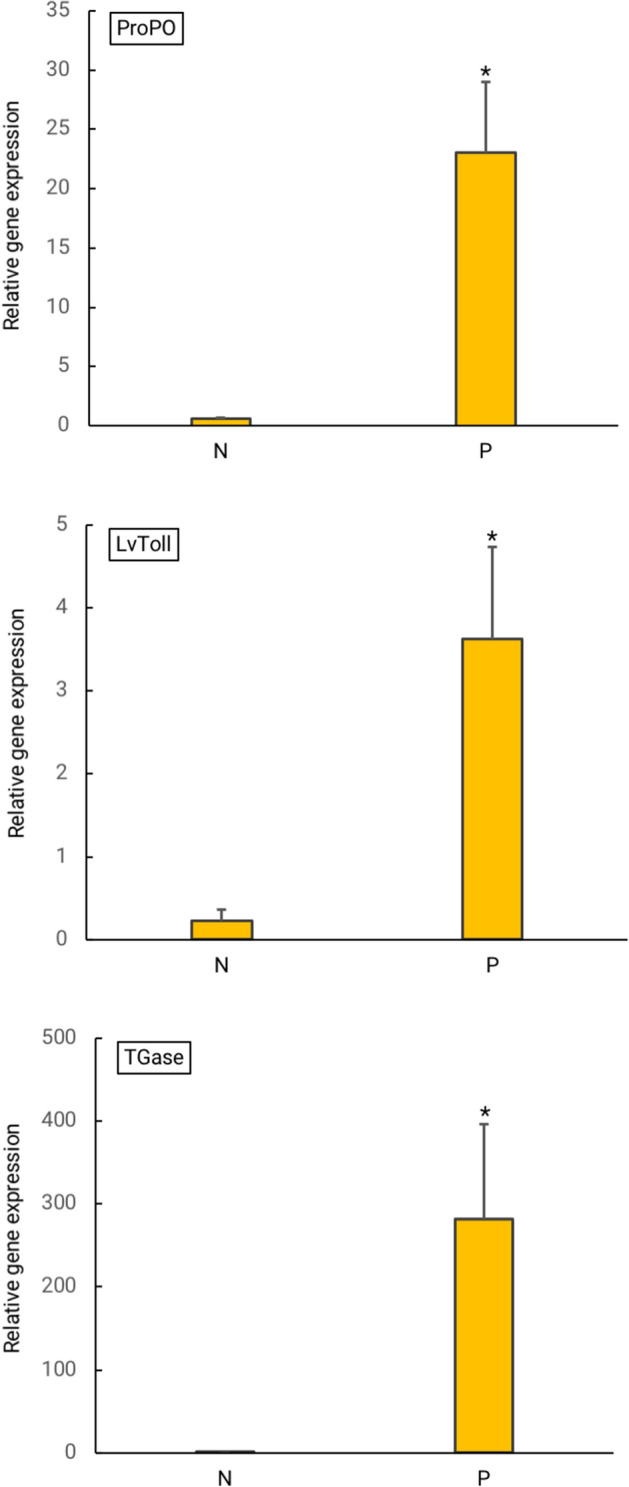
The relative expression levels of three immune-related genes (*proPO*, *LvToll* and *TGase*) between the probiotic treatment group (P) and the control group (N). Error bars indicate standard deviations (n = 3). Asterisks show a significant differences between the control group and probiotic treatment group (*p* < 0.05).

**Figure 5:**
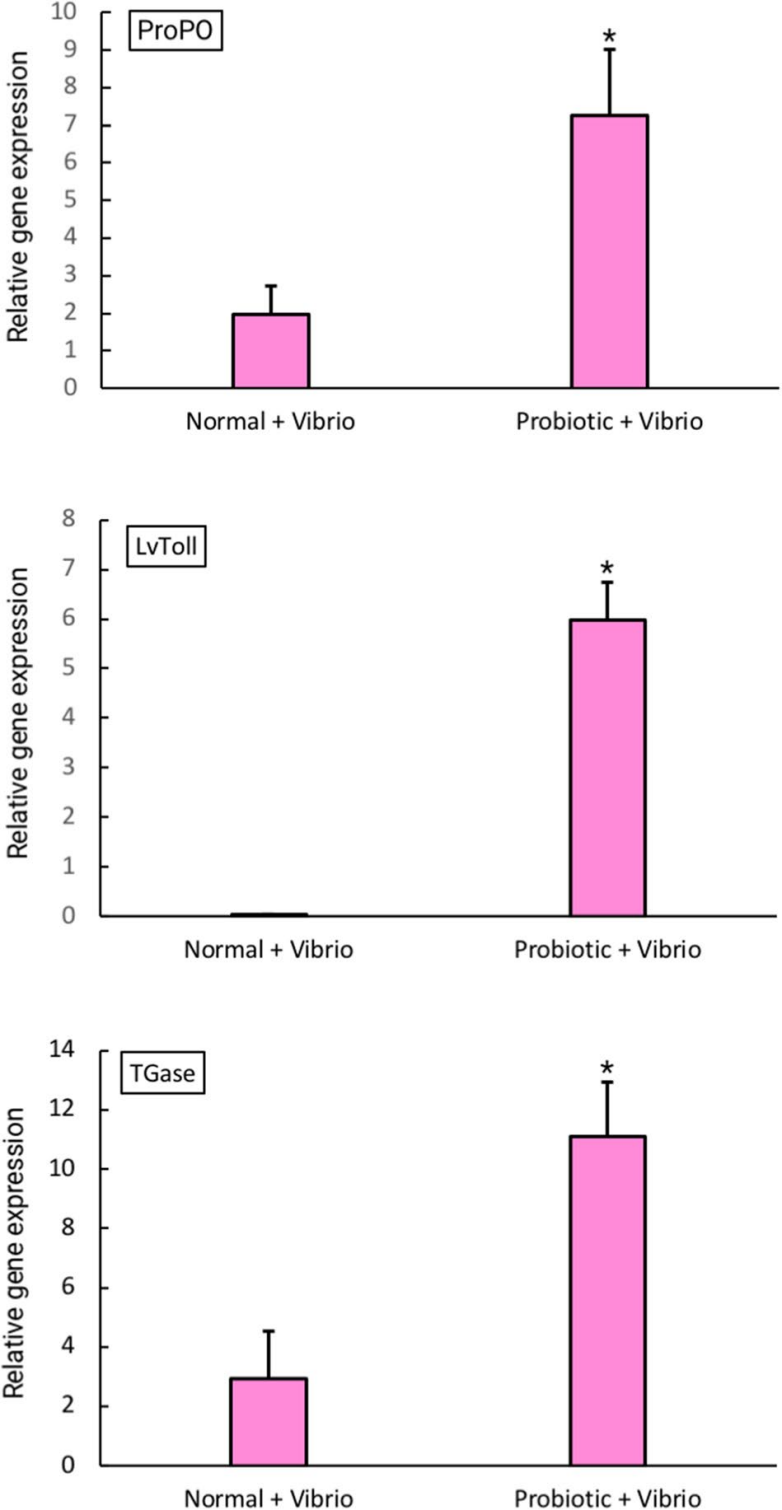
The relative expression levels of the three immune-related genes including *proPO*, *LvToll* and *TGase* from shrimp fed a diet supplemented with MR001 and challenged with *V. parahaemolyticus.* Error bars indicate standard deviations (n = 3). Asterisks show a significant differences between the control group (normal + vibrio) and probiotic treatment group (probiotic + vibrio) (*p* < 0.05).

To investigate the effects of experimental diet on the immunity of *L. vannamei*, immune-related gene expression in the hemocytes was studied. Three immune-related genes (*proPO*, *LvToll*, and *TGase*) were significantly upregulated in the probiotic treatment group compared to the control group (Fig. 4). In addition, Fig. 5 shows that after 48 h of *V. parahaemolyticus* challenge, the expression levels of *proPO*, *Lvtoll,* and *TGase* in probiotic feeding group were higher than that of the control group (commercial diet + vibrio).

### Genome features of *P. pentosaceus* MR001

**Figure 6:**
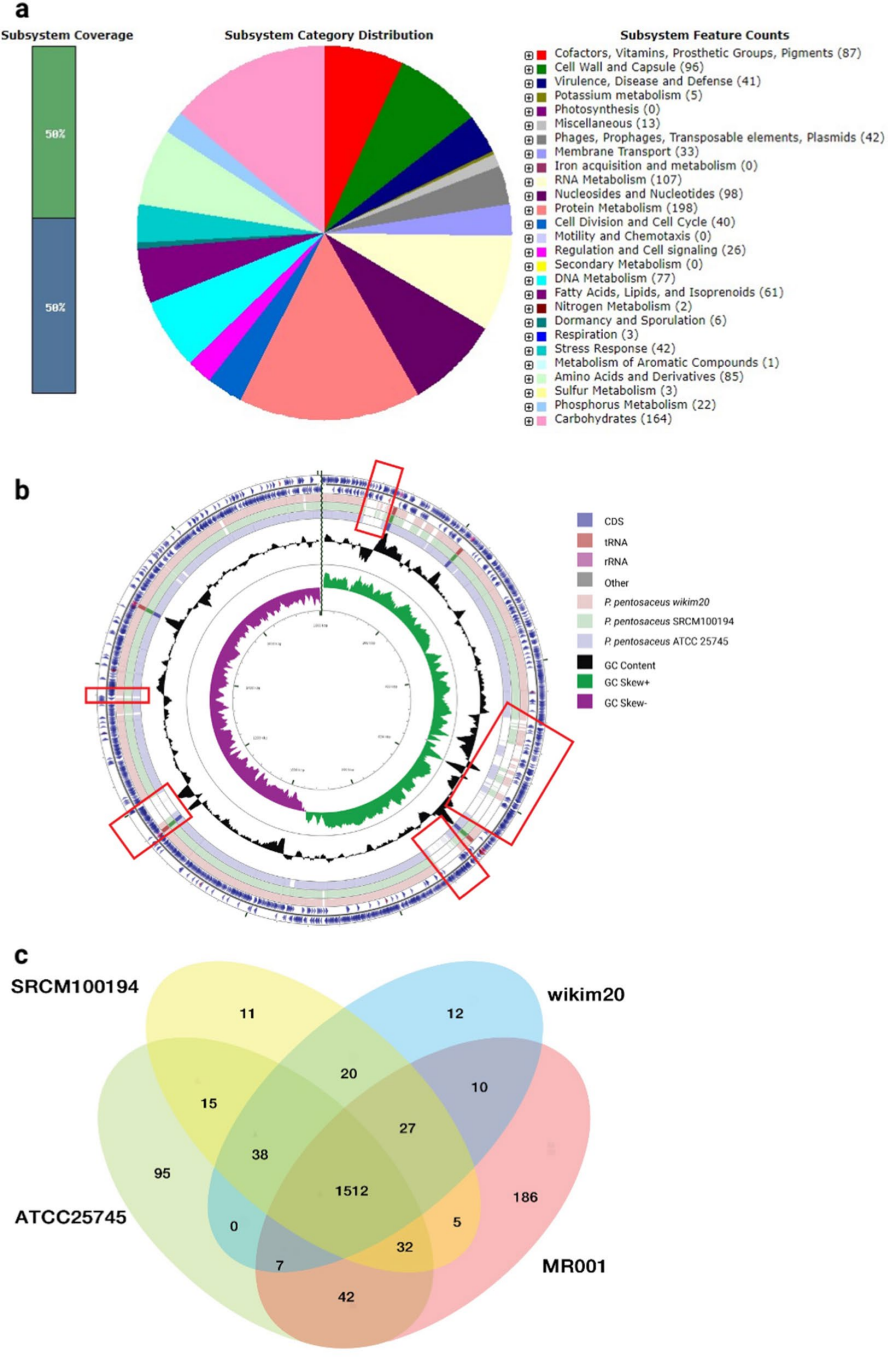
The annotation and comparison of *P. pentosaceus* MR001: (**a**) The distribution of the genes associated with the 25 COG functional categories/subsystems. (**b**) Comparative genomics against top 3 most closely related organisms. (**c**) A Venn diagram showing numbers of genes in common among the four species.

The genome of *P. pentosaceus* was sequenced using a hybrid next-generation sequencing (NGS) platform (Illumina Hiseq 2500 and PacBio RS II). De novo assembly generated a single complete genome. The full genome of *P. pentosaceus* MR001 consists of 1,804,896 nucleotides in one contig. The complete genome sequence then was annotated with the Rapid Annotations using Subsystem Technology (RAST) server version 2.0. The GC content of MR001 was 37.2%. In the main chromosome, 1,789 protein-coding genes were predicted. These genes were identified in 280 subsystems (Fig. 6a). This MR001 genome has 51 tRNAs and 15 rRNAs. Unlike *P. pentosaceus* wikim20 which had three circular plasmids, *P. pentosaceus* MR001 did not have any circular ones. The properties of the genome are shown in Table 7. *P. pentosaceus* MR001 was highly conserved when compared with *P. pentosaceus* ATCC25745, SRCM100194, and wikim20 (99% identity with 85–87% query coverage) (Supporting Table 2).

**Table 7:** Genomic nucleotide content and gene counts of *P. pentosaceus* MR001.

Attribute	Value
Size (bp)	1,804,896
GC content (%)	37.2
L50	1
Number of Contigs	1
Number of Subsystems	280
Number of Coding Sequences	1789
Number of rRNA	15
Number of tRNA	51

### Comparative genome analysis

A comparative genomics assay of MR001 with the three closest organisms revealed five unique regions that might produce their specific probiotic activity (Fig. 6b). Thirty three proteins encoded by *P. pentosaceus* MR001 genes were not detected or had sequence similarities of less than 50% in the comparative analysis with the three known strains, *P. pentosa*ceus ATCC25745, SRCM100194 and wikim20. Among these proteins, 239 hypothetical proteins with no clear functions were not further analyzed; the other 33 proteins are listed in Supporting Table 3.

We also identified orthologous proteins among the strains closest to *P. pentosaceus* MR001. Using a reciprocal all versus all BLAST search against ATCC25745, SRCM100194 and wikim20, we found that 1512 proteins formed the core set among these species. Based on the prediction of orthologous proteins, a four-set Venn diagram of the pan-genome among the species was included in Fig. 6c. *P. pentosaceus* MR001 contained a relatively higher number of 186 unique proteins than other species. The unique proteins were identified using BLASTP search against NR databases.

### Phylogenetic tree

**Figure 7:**
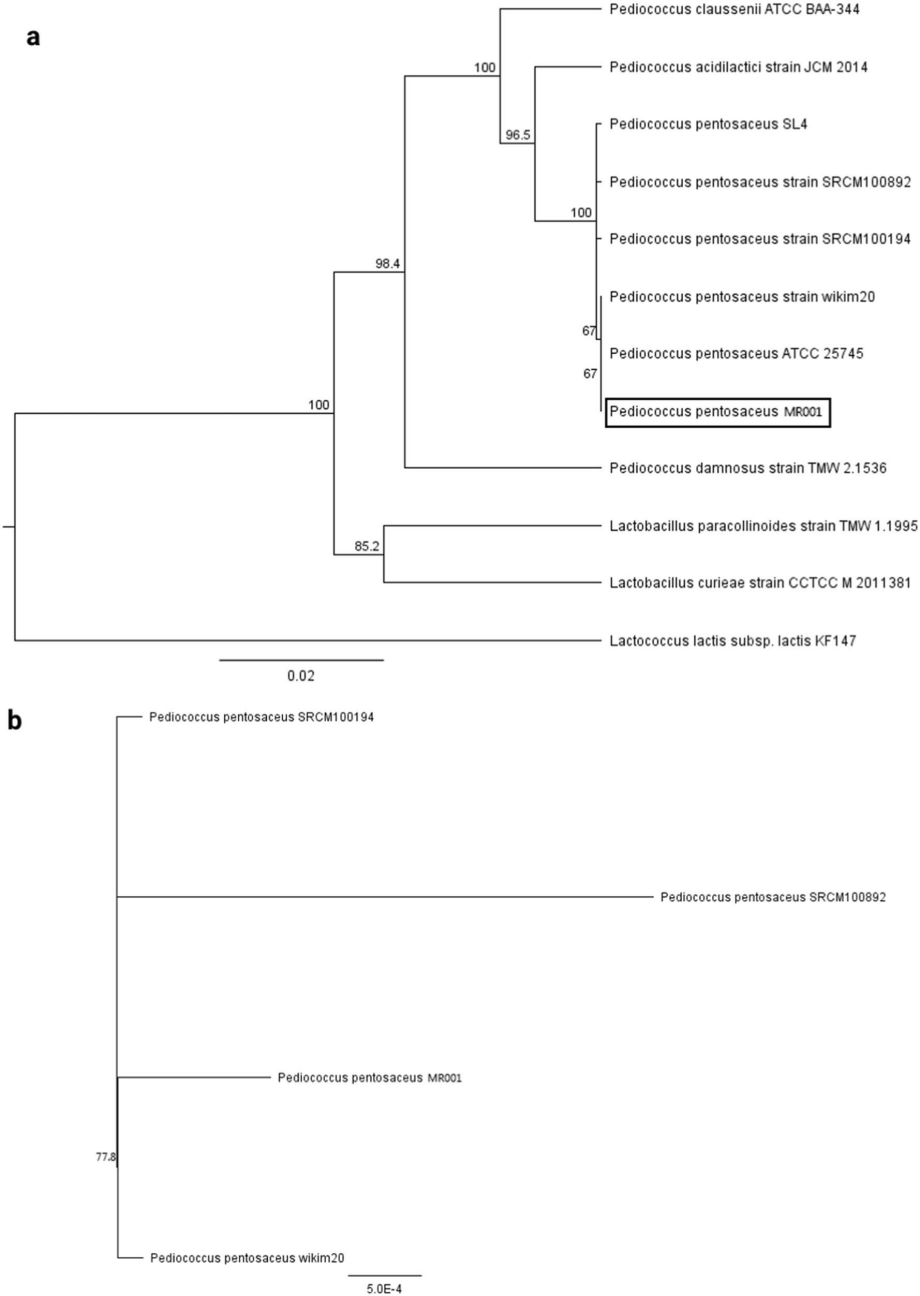
The phylogenetic tree of *P. pentosaceus* MR001 and its related species. (**a**) The multiple alignment of the 16S rRNA nucleotides was generated using MUSCLE. The phylogenetic tree was constructed with Geneious 9.1.2 using the neighbor-joining method with a bootstrap value of 1,000. (**b**) The phylogenetic tree highlighting the evolutionary relationships of the four strains of *P. pentosaceus* was based on concatenated nucleotide sequences of the *pheS*, *recA*, *tuF* and *gryA* genes (approximately 5800 bp).

A neighbor-joining tree based on the 16S rRNA gene sequence of strain MR001 showed the phylogenetic relationships among the species of the genus *Pediococcus*. *P. pentosaceus* formed a distinct branch separate from the groups of other members of the genus (Fig. 7a). Sequence analyses of *pheS*, *recA*, *tuF* and *gryA* housekeeping genes were carried out to definitively identify MR001, *P. pentosaceus* wikim20, *P. pentosaceus* SRCM100194 and *P. pentosaceus* SRCM100892. The results showed that the combination of the above housekeeping genes provided good phylogenetic relationships among the four strains. *P. pentosaceus* wikim20 was the closest evolutionary relative of strain MR001 (Fig. 7b).

### Genome analysis of *P. pentosaceus* MR001 exhibit as probiotic

We performed genomic data analysis to obtain a comprehensive view of relevant probiotic potency for the ability of probiotic strains in shrimp aquaculture. In *P. pentosaceus* MR001, we found that sortase A which recognizes the LPXTG motif in bacterial cell wall protein and catalyzes a cell wall sorting reaction and may play an important role in cell wall adherence.

In addition, *P. pentosaceus* MR001 has a gene that encodes choloylglycine hydrolase—an enzyme that participates in bile acid synthesis and gives microorganisms a bile salt resistance property. The existence of this gene is normally used as one of the criteria for probiotic strain selection. The *P. pentosaceus* MR001 genome also carried various coding proteins, including the chaperones DnaK and DnaJ, and the heat shock protein GrpE. Also, the coding sequence linked to antibiotic and toxic compound resistance was identified.

This genome showed unique coding sequences that include (1) copper chaperone, (2) copper translocating P-type ATPase and a negative transcriptional regulator-copper transport operon for copper homeostasis, (3) DNA gyrase subunits A and B, (4) topoisomerase IV subunits A and B, 5) the efflux pump Lde for resistance to fluoroquinolones, (6) the transcriptional regulator MerR family, (7) a DNA binding heavy metal response regulator, and (8) cadmium-transporting ATPase for cobalt-zinc cadmium resistance. In addition, colicin V, a peptide antibiotic that kills sensitive cells by disrupting their membrane potentials^13,14^, was found in *P. pentosaceus* MR001 strains. We also found protein-coding genes involved in oxidative stress tolerance. These include (1) ferroxidase which aids iron homeostasis, (2) the iron-binding ferritin-like antioxidant protein which can prevent DNA damage by reducing the formation of hydroxyl radicals^15^, (3) the peroxide stress regulator PerR which is a ferric uptake repressor-like protein involved in adaptation to oxidative stress and iron homeostasis, and (4) glutaredoxin which catalyzes glutathione-dependent disulfide reductions^16^. Increased oxidative stress has been shown to be related to various diseases in shrimp^17^. The results suggest that *P. pentosaceus* MR001 might effective at protecting cells against oxidative stress.

### Bacteriocin identification

In the preliminary investigation, *P. pentosaceus* MR001 exhibits an antagonist activity against *Vibrio* spp. We analyzed bacteriocins from the *P. pentosaceus* genome by performing a BLAST search against the bacteriocin database of Lactobacilli. The BLASTX parameters used for the identification included an E-value cutoff-point at 1e−4, and identity percentage at 30%. From the results, entrolysin A belonging to class III bacteriocin was identified with 54.84% identity (E-value = 2e−17).

### Gene expression of *sortase A* and *entrolysin A*

For determining the *sortase A* and *entrolysin A* gene were transcribed in *P. pentosaceus* MR001, We analyzed the transcription of these two genes by RT-PCR. We found that the full length of *sortase A* is 690 bp. This sequences is 98–100% identity with sortase A from *P. pentosaceus* isolated from other organisms and approximately 75–58% with *P. acidilactici*. Whereas a partial of *entrolysin A* gene was amplified and sequenced (Supporting Fig. 2). The sequence length of *entrolysin A* is 246 bp.

## Discussion

The development of non-antibiotic and environmentally friendly agents is a key factor for health management in aquaculture^1^. The application of probiotics in shrimp’s diet is beneficial to growth, immune response, and disease resistance. In this study, the genome analysis of *P. pentosaceus* MR001were performed to confirm the probiotic and antibiotic properties of the strain.

Growth performance is an important factor in shrimp aquaculture. We show that dietary supplementation with *P. pentosaceus* MR001 significantly improved the weight gain and growth rate of *L.vannamei*. Compared to the control group, the lower value of FCR observed in the group that received an MR001-supplemented diet suggests that *P. pentosaceus* MR001 could improve the feed utilization and growth performance of shrimp. Our findings are consistent with other studies that probiotic supplementation significantly increased growth performance and nutritional utilization in shrimp^18–21^.

The enhanced growth performance of shrimp in this work might have been due to increased digestive enzyme activity induced by *P. pentosaceus*. The shrimp digestive system is activated particularly in the larval and early post-larval stages when the probiotics would have the greatest effect^22^. The digestive enzyme activities of shrimp are important indicators of the organism’s ability to metabolize given nutrients. Trypsin amylase and lipase activity were significantly increased in the probiotic group compared with the control group (*p* < 0.05). A few studies of shrimp supplemented with probiotic *Pediococcus* spp.^18,23^ demonstrated similar responses to our results. The higher level of enzyme activity obtained with diets containing probiotics may help to improve the digestion of protein, starch and fat, which might in turn explain the better growth observed in the probiotic-supplemented shrimp. Carbohydrate and protein macronutrients can influence the activities of digestive enzyme, especially trypsin, in shrimp^24^. Therefore, the specific activity of trypsin and the T/C ratio have been used as indicators for growth and feed conversion efficiency in aquatic animals^25^. In this study, the T/C ratio appeared to be higher in the groups that received probiotic diets, although insignificant. It is possible that the T/C indicator would have shown significant differences either in larger treatment groups or with prolonged treatment^25^. Suggested by Won et al.^18^, improved growth performance through probiotic supplementation may be due to enhanced gastrointestinal performance^26,27^ and immune response^28,29^.

The intestine, regarded as the digestion and absorption center, produces the most sensitive tissue response to environmental stress in aquatic animals^30,31^. In this study, the intestine epithelium height increased in the probiotic feeding group. Increased height and/or density of intestinal epithelial cell microvilli and enterocytes has been demonstrated to provide a vast absorptive surface area and nutrient absorptive ability^31,32^.

The crustacean immune system eliminates pathogens very efficiently through humoral and cellular immune processes. The shrimp immune system works without a memory basis by activating a response using hemocytes that attack foreign cells during infection^33^. Immunostimulation is an alternative strategy and a vital method of alerting a shrimp’s defense system to increase resistance to various pathogens. It also indirectly enhances growth performance^19,31^. In this study, the expression of three immune-related genes was investigated using real-time PCR in order to evaluate the immune status of shrimp after supplementation with probiotics. The relative expression levels of the immune genes *proPO*, *LvToll* and *TGase* in hemocytes were significantly higher in *P. pentosaceus* MR001-supplemented shrimp than in control shrimp (*p* < 0.05).

proPO enzyme is a key component of the prophenoloxidase-activating system which plays an important role in the invertebrate immune system. Active proPO induces the oxidation of phenol to quinone and prompts the production of melanin, allowing a rapid response to pathogen infection. The upregulation of *proPO* in the probiotic feeding group may have enhanced resistance against pathogens. Similar results were found in other studies where greater upregulation of *proPO* was detected when pathogen-challenged shrimp were fed with probiotic diets^18,29^*.*

The Toll-like receptor (TLR) family is a highly conserved group of proteins that participate in innate immune responses^34^. In shrimp, Toll receptors are important pathogen recognition receptors (PRRs), playing a vital role in defending against viral and bacterial challenge^35^. From this study, we inferred that upregulation of *LvToll* receptor genes may increase pathogen recognition and increase disease response. A similar result was found in *L. vannamei* given mixed *Bacillus* spp.^36^.

The enzyme transglutaminase (TGase) is known to be involved in blood coagulation, a conserved defense mechanism among invertebrates^37^. In shrimp, an effective and efficient blood coagulation system increases the chances of survival, particularly in cases of injury^38^. Fagutao et al.^38^, reported that the absence of TGase rendered shrimp susceptible to both bacterial and viral infections, suggesting that TGase is an essential component of the immune system of shrimp.

It has been known that *V. parahaemolyticus* infection causes serious disease in *L. vannamei*. Our results show that shrimp treated with *P. pentosaceus* MR001 exhibited stronger disease resistance against *V. parahaemolyticus* than those in the control group. As noted in previous reports^10,18^, the mortality rate of shrimp infected with *Vibrio* spp. was lower in the *P. pentosaceus* treatment groups than in the control groups. In agreement with gene expression analysis, the relative expression levels of the immune-related genes (*proPO*, *LvToll* and *Tgase*) in hemocytes were also significantly upregulated in *P. pentosaceus* MR001-supplemented shrimp after 48 h post *V. parahaemolyticus* infection than in control group (*p* < 0.05). Our data imply that *P. pentosaceus* MR001 could enhance systemic innate immunity during the *Vibrio* challenge by stimulating immune gene production.

The genomic data analysis of *P. pentosaceus* strains showed a genome size and GC content of 1.8 Mbp and 37.2%, respectively. A total of 1789 sequences were assigned to putative functions, and the remainder could be classified as hypothetical proteins. Many of these functional proteins were potentially related to probiotic properties.

The ability to adhere to the gastrointestinal tract/mucosa is an important property of most probiotics^39,40^. In vitro analysis, MR001 showed strong auto-aggregative ability and high hydrophobicity (> 80%). Probiotic bacteria, especially LAB with aggregation ability and hydrophobic cell surface are more capable of adhering to intestinal epithelium^41^. Additionally, MR001 possessed strong co-aggregation with both shrimp pathogenes. The co-aggregation ability could play a significant role in inhibiting the growth of pathogenic strains in the GIT and can help prevent colonization by invading foodborne pathogens^42,43^.

Sortase A is one of the proteins encoded by *P. pentosaceus* MR001 and predicted to influence the adhesive potential of *P. pentosaceus.* Sortase A recognizes the sequence motif LPXTG in the cell wall proteins^44^. This protein has also been found in other probiotic bacteria such as *Lactobacillus plantarum*^45^ *Lactobacillus rhamnosus*^46^ and *Bifidobacterium bifidum*^47^. The genomes of most Gram-positive bacteria encoded two or more sortase enzymes in order to fulfilled different functions^44^.

Upon ingestion by the host and during transit through the gastrointestinal tract (GIT), probiotics encounter various environmental conditions. Firstly, they need to survive the harsh conditions of the stomach. Our analysis of this genome revealed bacterial responses to acid and bile, and other stress resistance mechanisms. This finding was in agreement with in vitro analysis of MR001, when it was able to tolerate a wide range of salt and bile salt concentrations and can expose the acidic condition (pH 3). Many other stressful conditions, such as oxidative stress, can also be encountered in the GIT. A number of stress proteins that regulate the adaptation of this strain to GIT stress were found in this study, in particular, the chaperones that are known to intervene in response to numerous stresses. These proteins are involved in important tasks such as protein folding, renaturation, protection of denatured proteins, and removal of damaged proteins^48^. Important molecular chaperones include DnaK, the well-known heat shock protein related to acid adaptation^49^, which was found in this study. Using a microarray analysis, Pfeiler et al.^50^ found that *dnaK* and *grpE* were upregulated in *L. acidophilus* NCFM after exposure to bile^50^. Heat shock proteins appear to be especially pivotal for long-term acid stress resistance.

Due to in antibacterial assay, we found that MR001 can inhibit shrimp pathogens. Thus, the bacteriocins were analysed in genome of MR001. Bacteriocins are a group of potent antimicrobial peptides. Most bacteria, including LAB, can produce at least one bacteriocin^51^. The production of bacteriocins depends on the microbial strain and culture condition^52^. Some bacteriocins are used as alternatives to antibiotics and were deployed in the food industry and medicine^53–55^. In this study, entrolysin A, a type III bacteriocin, was found in genome by in silico analysis. Entrolysin A is a cell wall-degrading bacteriocin first reported to be produced by *Enterococcus faecalis* isolated from fish^56^. It can inhibit the growth of various Gram-positive bacteria such as enterococci, pediococci, and lactococci. Our findings are in agreement with that of Jiang et al.^57^, who comparatively studied the genomes of 65 *P. pentosaceus* strains isolated from food, humans, and animals. They found four kinds of bacteriocins in these strains; two of which, enterolysin A and Bac, had not been previously reported in this species.

In this study, entrolysin A was amplified to confirm that it is transcribed in the MR001 strain. However, just a small part of this gene could be amplified. This phenomenon may have been caused by the integration of some mobile DNA such as prophages, mobile genetic elements, and insertion elements. In the comparative genome analysis, among 33 proteins, two insertion sequences (IS) were found (IS6 and IS1182), revealing the mechanism of MR001 adaptation for survival via integration of these elements to its genome^58^. Additionally, four sugar transport proteins were detected only in MR001; three were PTS system fructose-specific EIIABC components (FruA) and one a PTS system oligo-beta mannoside-specific EIIC component (gmuC). In bacteria, to carry out its catalytic function in sugar transport and phosphorylation, the phosphotransferase system (PTS) uses the phosphoenolpyruvate (PEP) as an energy source and phosphoryl donor. The phosphoryl group of PEP is usually transferred via four distinct proteins to the transported sugar that is bound to the respective membrane components of the PTS^59^. In general the regulatory roles of protein components of the PTS for the control of carbohydrate metabolism have been reported. Notably, the PTS also fulfils numerous roles in the regulation of biofilm formation, stress response, gut colonization, chemotaxis, and virulence^60–64^. Various types of sugar transport systems may designed to specific probiotic-prebiotic in the future.

## Conclusion

This study showed that the use of *P. pentosaceus* MR001 as a dietary probiotic for the white shrimp, *L. vannamei*, significantly improved growth performance, feed utilization, digestive enzyme activities, and disease resistance against vibriosis. A probiotic supplement of *P. pentosaceus* MR001 at 10^9^ CFU/g in the diet of white shrimp is recommended. Our elucidation of the genome sequence of *P. pentosaceus* MR001 allows for a deeper understanding of the strain’s probiotic potential, facilitating the future development of food additives for marine aquaculture.

## Materials and methods

### Candidate probiotic isolation

Guts of 3 animals were removed from *Macrobrachium rosenbergii.* Each gut was dissected aseptically and homogenized in an eppendorf tube with 200 μL of sterile saline solution with 0.85% NaCl. Homogenates were serially diluted (10^–1^–10^–5^) with 0.85% NaCl, and 1 mL of diluted homogenate was poured on de Man, Rogosa and Sharpe (MRS) agar plates and incubated at 30 °C for 24 h. All isolates were examined against *V. harveyi* using the agar well diffusion assay to test the antagonistic ability using method below. Colonies producing inhibition zones were isolated and future tested for the hydrophobicity using the microbial adhesion to solvents (*p*-xylene) following with slight modification was used to determine cell surface properties^43^. The isolates that showed the highest antibacterial activity against pathogenic *V. harveyi* and high hydrophobicity were characterized by Gram staining and catalase test.

Total DNA of isolate was extracted from a colony using a PureLink® Genomic DNA (Invitrogen) according to the manufacturer’s instructions. The amplification of 16S ribosomal DNA was performed by single PCR using the universal primers 27f. and 1492r (Supporting Table 4).

### In vitro characterization of *P. pentosaceus* MR001

#### Antibacterial activity

The isolates were re-examined against *V. harveyi* and *V. parahaemolyticus* using the agar well diffusion assay to confirm the antagonistic ability. All isolates were grown in MRS broth at 30 °C for 24 h. After incubation, the supernatant was collected by centrifugation (10,000 rpm; 1 min) and re-suspended into 100 µl of MRS medium. The target Vibrio strains were grown overnight in 10 ml of Trypticase Soy Broth (TSB) at 30 °C, and 1 ml (10^8^ CFU/mL) of each Vibrio was mixed with 20 ml of melted TSA supplemented with 2% NaCl. Wells (6 mm) were then punched into the agar and 100 µl of isolates supernatants were added. Plates were incubated at 30 °C and observed for clearing zones around the wells after 24 h. Sterile MRS broth was used as the negative control and 100 μg/ml of tretacycline was used as the positive control (P).

#### Acid resistance

Isolated strains were cultivated in MRS broth until the OD600 reached 1.2. One ml of culture was centrifuged at 12,000 g for 10 min at 4 °C and cell pellet was resuspended in MRS broth where the pH was adjusted to 3 and 7 (control), respectively, by 1 N HCl. Cell suspensions were incubated for 3 h at 37 °C and then viable cells were counted by standard plate counting. Measurements were done in triplicates and the mean values were shown.

#### Bile and NaCl tolerance

The bile tolerance of *Pediococcus pentosaceus* was determined using a modified version of a previously method^65^. Briefly, bile salt tolerance test was determined by inoculating *P*. *pentosaceus* MR001 into various MRS broth containing 0.6%, 0.8% and 1.0% bile salts (Sigma-Aldrich). The suspension was incubated for 6 h at 30$^\circ$C. After incubation, the suspensions were serially diluted in sterile PBS. 100 μL of the suspension was flooded in MRS agar with triplicate and incubated for 24–48 h at 37$^\circ$C and the viable colony that appears was counted using colony counter.

To determine NaCl tolerance, *P*. *pentosaceus* MR001 was grown in MRS broth supplemented with different concentrations of NaCl (1–6%). 0.1 mL of overnight culture was inoculated into 10 mL of MRS broth with various percentage of NaCl. After 3 h of incubation at 37 °C, MR001 was sub-cultured in MRS agar and was incubated at 37 °C for 24 to 48 h. The NaCl tolerance of MR001 was calculated by counting viable cells on plates.

#### Auto-aggregation and co-aggregation assay

The specific cell–cell interactions were determined using auto-aggregation assay^66^ and co-aggregation assay^65^ with slightly modified methods. *P. pentosaceus* was cultured at 30 ºC for 20 h in MRS medium. The bacterial cells were harvested at 5000 g for 10 min at room temperature, washed with PBS and resuspended in PBS to 10^8^ CFU/ml. For the auto-aggregation assay, 4 ml of each bacterial suspension were vortexed for 10 s and incubated at 37 °C for 2 h, 6 h and 24 h. The absorbance of the supernatant was measured at 600 nm. The auto-aggregation percentage was expressed as: 1 − (A_t_/A_0_) × 100, where A_0_ represents the absorbance at t = 0, and A_t_ represents the absorbance at incubation time.

For the co-aggregation assay, *Vibrio spp.* were cultured in TSA medium + 1.5%NaCl at 37 ºC for 16 h. Equal volumes of cell suspensions (10^8^ CFU/ml) were mixed, vortexed for 10 s and incubated at 30 ºC and 37 ºC for 4 h without agitation. The absorbance (A 600 nm) of the mixture was determined during incubation at 2 h, 6 h and 24 h. The percentage of co-aggregation was calculated following the formula [(A_vibio_ + A_pediococcus_)/2 − (A_mix_)/(A_vibio_ + A_pediococcus_)/2] × 100, where A_vibio_ and A_pediococcus_ represent the A600 of individual bacterial suspensions and A_mix_ represents the absorbance of their mixture after incubation for different times.

### Samples for feeding experiments

Healthy specimens of *L. vannamei* were provided by Kanjana Farm in Nakhon Si Thammarat province, Thailand. They were placed in an indoor cement pond and cultured for one week in filtered, aerated seawater (salinity 30%, pH 8.5). Shrimp (average weight 4–5 g) were selected for experiment and randomly divided into four groups with three replicates for growth rate experiments and two groups with three replicates for survival rate experiments.

### Preparation of shrimp feed supplemented with *P. pentosaceus* MR001

*P. pentosaceus* MR001 was cultured and incubated at 30$^\circ \mathrm{C}$ for 20 h in MRS broth (HIMEDIA) on a shaker at 200 rpm to a final concentration of about 10^10^ CFU/ml. The probiotic was harvested by centrifugation and washed twice with sterile saline and mixed with commercial shrimp feed in the ratio of 1 ml of probiotic to 1 g of shrimp feed. The probiotic concentrations of the final suspensions in the commercial feed were 1 × 10^7^, 10^8^ and 10^9^ CFU/g. After feed preparation, MR001 concentrations in the diet were confirmed by a plating technique in which a sample of each feed was serially diluted in MRS broth. New batches of feed were produced every week to keep up *P. pentosaceus* MR001 viability.

### Determination of *L. vannamei* growth rate

The shrimp were divided into four treatment groups (three tanks per treatment, 15 shrimp per tank). All shrimp in each tank were initially fed twice a day, each time with a diet weighing 10% of their total body weight. At the beginning of the experiment, five specimens were randomly collected from each group to measure their initial body weight (W_0_). After three weeks of treatment with the probiotic supplement, five specimens were selected at random from each group to measure their final body weight (Wt). The weight gain rate of each group was calculated following the formula [Weight gain (%) = 100 $\times$ (Wt− W_0_)/W_0_]. Other biological performance criteria such as specific growth rate (SRG), feed efficiency, and feed convention ration (FCR) were also determined.

### Determination of intestinal digestive enzyme activity

Sampling was conducted at day 14 of probiotic supplementation. Five shrimp were randomly selected from each group. Shrimp gastrointestinal tracts were removed and homogenized in ice-cold deionized water (1:3) using a micro-homogenizer. The homogenate was centrifuged at 15,000 g for 30 min at 4 $^\circ{\rm C}$. The supernatant was kept at -20 ℃ until used. Total protein was evaluated using a BSA standard curve as described by Lowry et al.^67^. The activity of the total protein was measured by a spectrophotometer at 750 nm. Trypsin and chymotrypsin were determined based on the method of Rungruangsak–Torrissen et al.^68^. The enzymes were incubated at 50 ℃ for 10 min by methods developed by Gamboa-Delgado et al.^24^. The activity of these two enzymes was measured spectrophotometrically at 410 nm, and the comparison was made with the linear response concentration range of a p-nitroanilide standard. Amylase^69^ and cellulase^70^ activity were determined using a starch solution and carboxymethylcellulose (CMC) as respective substrates, respectively. The optimal condition of incubation was 55 ℃ for 10 min, following the method of Xue et al.^70^. Both enzymes were then evaluated spectrophotometrically at 540 nm and compared with maltose and glucose standard curves, respectively. Lipase activity was measured in triplicate by the method of Stuckmann & Winker^71^. Lipase was detected spectrophotometrically at 410 nm based on the cleavage of p-nitrophenyl palmitate (p-NPP; Sigma). The optimal condition used was at pH 8 and 60 ℃.

### Intestinal Histology

Shrimp intestinal tissue was fixed in 4% neutral buffered formaldehyde and then dehydrated in a graded ethanol series and embedded in paraffin. Tissue blocks were sectioned 4 μm thick and stained with hematoxylin and eosin (H&E). Villi heights were then evaluated and observed under a light microscope. At least 5 fields were observed in each samples.

### Survivability test

To determine the effect of *P. pentosaceus* MR001 on the survival rate of *L. vannamei* post-larvae, the shrimp were divided into two groups (control group and probiotic group; 15 shrimp per group). At the end of the three-week feeding trial, shrimp fed with MR001 supplemented and non-supplemented diets were exposed to pathogenic *V. parahaemolyticus* at a level of 10^5^ CFU/ml (LD50) by adding the bacteria to the water for 24 h. During the first 24 h post-infection, water was not renewed to ensure the infection. All groups were fed a basal diet and kept under observation for 10 days. At the end of the feeding trial, post-larvae survival rates and the numbers of dead shrimp per each replicate were calculated and recorded. The survival rate (SR) of each group was calculated as SR (%) = [final number/initial number] × 100.

### Immune-related gene expression analysis

At the end of three week feeding trial, five shrimp per experimental diet from each tank were collected (3 tanks per experimental diet) for RNA extraction. In addition, to determine immune-gene expression after pathogen infection, shrimp feeding with each diet were injected with *V. parahaemolyticus.* Then 24 h after *V. parahaemolyticus* challenge, shrimp were sampled for determination of immune-related genes expression. RNA was extracted using TRIzol reagent (Invitrogen) following the standard protocol and RNA with an absorbance ratios (A260/A280) greater than 1.8 was used for the next step in which cDNA was synthesized from 1 µg RNA with a Viva cDNA Synthesis Kit (Vivantis Technologies Sdn. Bhd.). The transcriptional expression levels of *proPO*, *LvToll* and *TGase* genes were determined by real-time quantitative PCR (RT-qPCR) using the SensiFAST SYBR NO-ROX kit in accordance with the manufacturer’s protocol (Bioline). RT-qPCR was performed in the following order: denaturation at 95 ℃ for 5 min and then 40 cycles of 95 ℃ for 30 s, 55–58 ℃ (depending on each primer) for 30 s, and 72 ℃ for 30 s. A dissociation curve analysis was performed at the end of qPCR to confirm the specificity of the PCR products. The *EF1* gene of *L. vannamei* was used as an internal control to verify the successful reverse transcription and to calibrate cDNA template. Primer sequences were presented in Supporting Table 4.

### Statistical analysis

The data were analyzed using IBM SPSS version 24. One-way analysis of variance was used to determine significant variations between the treatments. The differences between means were evaluated and compared by post hoc multiple comparison test (Least Significant Difference, LSD). All results were regarded as significant when *p* < 0.05. Data were reported as means ± standard deviations.

### Genomic DNA isolation and genome sequencing

DNA extraction was performed with a PureLink genomic DNA mini kit (Invitrogen, USA) following the manufacturer’s instructions. The isolated DNA concentration was quantified using the Qubit fluoromete (Life Technologies, USA). DNA integrity was checked using gel electrophoresis in1% agarose gel. NGS of genomic DNA from *P. pentosaceus* MR001 was conducted using the TruSeq DNA PCR-Free kit library on the HiSeq 2500 platform. Total read bases were 973.6 Mbp with 9,639,394 total reads. The GC content was 37.05%, and Q30 was 92.91%. After filtering, the total number of bases, reads, GC (%) and Q30 (%) were 853.7 Mbp, 8,568,054 reads, 37.01% and 97.84%, respectively. Third-generation DNA sequencing was performed with the PacBio RS II technology using a PacBio P6C4 chemistry sequencing kit. One single-molecule real-time (SMRT) cell yielded 128,058 subreads (1,123,882,312 bp) with an average read length of approximately 8.76 kbp.

### Genome assembly

**Figure 8:**
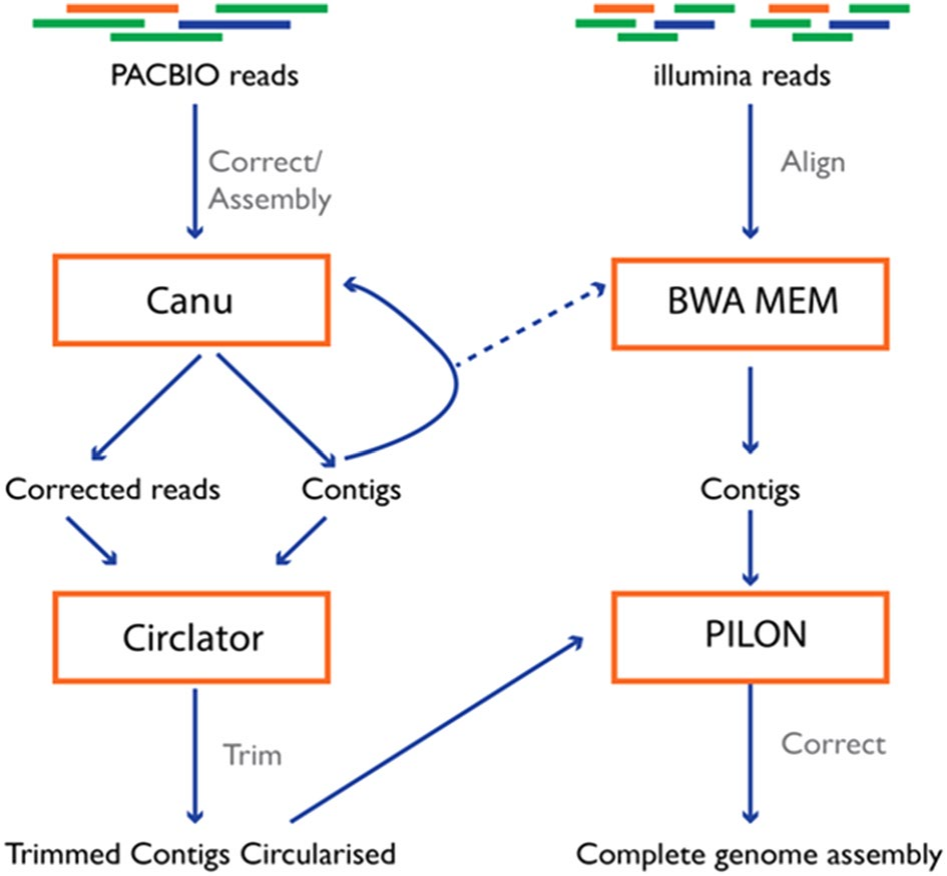
Genome Assembly Workflow.

We used the software called Canu to assembly high-noise single-molecule sequencing from PACBIO. The default parameter of the software was set to use with the *P. pentosaceus* MR001 with setting the genome size around 2 Mbp. The first result from the software returned 1,836,985 on genome size and 37.2%GC. There is no sign of any plasmid in this strain. Then, we use the Circlator to identify and trim overhangs and orients the start position at an appropriate gene by using assembly contigs and the corrected reads prepared by Canu version 2.1^72^ with default parameters of the software. The contig length was trimmed to 1,804,890 bp. Finally, we corrected the PACBIO assembly with Illumina reads. The Illumina reads were aligned to the draft assembly using BWA^73^, BWA-mem , and SAMTOOLS version 1.7^74^ Then, Pilon version 1.24^75^ was used to automatically improve draft assemblies and find variation among strains, including large event detection. The result was still the same result from circularization. There was no any change in the result. The assembly workflow is illustrated in Fig. 8.

### Genome annotation

Glimmer 3^76^ and Genemark software^77–79^ were used to identify genes in the genome. Functional annotation was achieved using RAST (Rapid Annotation using Subsystem Technology)^80,81^. tRNA was predicted by tRNAscan-SE 1.21^82–84^ and rRNA genes were predicted by RNAmmer 1.2^85^. All the predicted proteins were used in the BLASTP search against the NCBI non-redundant (nr) protein database to find homologs. Prophage regions were predicted using the PHAge Search Tool (PHAST) webserver^86^. Regions of clustered regularly interspaced short palindromic repeats (CRISPR) were searched using the CRISPRFinder server^87^.

### Genome comparisons and visualization

The microbial nucleotide BLAST database was queried to find the closest relatives of *P. pentosaceus* MR001 for comparison. *P. pentosaceus* wikim20 (NZ_CP015918.1), *P. pentosaceus* SRCM100194 (NZ_CP021927.1), *P. pentosaceus* ATCC 25,745 (NC_008525.1), *P. pentosaceus* SL4 (NC_022780.1), and *P. pentosaceus* SRCM100892 (NZ_CP021474.1) were the top five BLAST hits with approximately 99% identity and very significant E-values. The similarity of MR001 to its three closest relatives was analyzed and visualized using the CGView Server, with the E-value cutoff set to 0.0001 and the identity cutoff set to 30%, based on the BLASTX comparison. The circular representation of the chromosome of *P. pentosaceus* was produced using the CGView Server V1.0^88^. Subsystem mapping was produced by RAST version 2.0^80,81^. In addition, a circular representation of the plasmids was drawn using Geneious 9.1.2^89^.

### Bacteriocin identification

Bacteriocin identification was analyzed following the procedure of Surachat et al., 2017^90^. The nucleotide sequence of *P. pentosaceus* was first screened against the *BACTERIOCINS* database using BLASTX. The preliminary screening result was then generated and used as the target of a search for similar protein sequences from *Lactobacilli* in the NCBI and UniProt databases. After removing data redundancy, the local database was then created. Finally, the preliminary result from the first BLAST was used as query sequences to search against the newly created database using BLASTX with the E-value set sequence and confirmed as a bacteriocin in *P. pentosaceus.* We identified the bacteriocin using the Bagel4 server. We also used the Bagel database^91^ (Class I, II, III) in the BLAST analysis to identify antimicrobial proteins. The *P. pentosaceus* MR001 sequence was then used in a BLASTX search with the E-value and %identity cutoff set to 10^−4^ and 30%.

### Phylogenetic tree analysis

16S rRNA sequences (approximately 1,580 bp) were obtained from the following 11 species: *P. pentosaceus* ATCC 25,745, *P. pentosaceus* SL4, *P. pentosaceus* SRCM100194, *P. pentosaceus* SRCM100892, *P. pentosaceus* wikim20, *P. acidilactici* JCM 2014, *P. claussenii* ATCC BAA-344, *P. damnosus* TMW 2.1536, *L. curieae* CCTCC M 2,011,381, *L. paracollinoides* TMW 1.1995, and *Lactococcus lactis subsp. lactis KF147*. The multiple alignment of 16sRNA was generated using MUSCLE^92^. The phylogenetic tree was constructed with the Geneiuos 9.1.2^89^ program using the neighbour-joining method with a bootstrap value of 1,000. The phylogenetic tree highlighting the evolutionary relationships of the four strains of *P. pentosaceus* was based on concatenated nucleotide sequences of the pheS, recA, tuF and gryA genes (approximately 5800 bp).

### The amplification of *sortase A* and *entrolysin A* transcripts

*P. pentosaceus* culture (at an optical density at 600 nm of 0.5) were centrifuged at 4000 g for 10 min, and the pellet was resuspended in 1 ml of Trizol (Invitrogen). Bacteria were broken once for 30 s in the Bead Beater at maximum speed. The supernatants was then extracted with chloroform : isoamyl alcohol. Total RNA was precipitated using isopropanol. The contaminating DNA was treated with RNase-Free DNase I (Roche). First-strand cDNA was synthesized from 1 µg of total RNA using the SuperScript III First-Strand Synthesis System.

The transcripts of *sortase A* and *entrolysin A* were analysed by PCR using the each specific primers SrtA-F and SrtA-R for *sortase A* and EntA-F and EntA-R for *entrolysin A*. The thermal cycling profile used was 95 °C for 3 min, followed by 30 cycles of 95 °C for 30 s, annealing for 30 s at 52 °C for sortase A and 60 °C for entrolysin A and 72 °C for 30 s. PCR products were analysed on 1.5% agarose gel.

## Supplementary Information

Supplementary Information.

## Data Availability

This Whole Genome project has been deposited in the NCBI BioProject, BioSample, and GenBank under the following respective accession numbers: PRJNA596088, SAMN13612138, and CP047081. The raw sequence PacBio and Illumina reads have been deposited in the SRA database (accession number: SRR10717635-36).
