# Supplementary material for: Evaluation of probiotic characteristics and whole genome analysis of Pediococcus pentosaceus MR001 for use as probiotic bacteria in shrimp aquaculture
Source: Sci Rep. 2021 Sep 15;11:18334. doi: 10.1038/s41598-021-96780-z (PMC8443617; doi:10.1038/s41598-021-96780-z)
Supplement: Supplementary file 1 — Supplementary Information. [file 41598_2021_96780_MOESM1_ESM.pdf]

Evaluation of Probiotic Characteristics and Whole Genome Analysis of *Pediococcus pentosaceus* MR001 for use as probiotic bacteria in shrimp aquaculture.

Warapond Wanna[1,2*], Komwit Surachat[4,5], Panmile Kaitimonchai[1], Amornrat Phongdara[2,3]

[1]*Division of Biological Science, Faculty of Science, Prince of Songkla University, Hat Yai, Songkhla 90110, Thailand*

[2]*Center for Genomics and Bioinformatics Research, Faculty of Science, Prince of Songkla University, Songkhla, 90110, Thailand*

[3]*Faculty of Medical Technology, Prince of Songkla University, Songkhla, 90110, Thailand*

[4]*Division of Computational Science, Faculty of Science, Prince of Songkla University, Songkhla, 90110, Thailand*

[5]*Molecular Evolution and Computational Biology Research Unit, Faculty of Science, Prince of Songkla University, Hat Yai, Songkhla, 90110, Thailand*

*****Corresponding author, Email address**:** w**.**warapond@gmail**.**com

Clone no.	Inhibition zone	hydrophobicity
Iso1	14.33±0.47	57.33±5.44
Iso2	15.00±0.82	28.00±2.16
Iso3	15.50±0.41	44.00±3.74
Iso4	18.67±0.47	90.00±2.16
Iso5	15.56±0.47	29.67±0.47
Iso6	16.00±0.82	38.33±3.68
Iso7	16.33±0.47	60.66±1.70
Iso8	18.33±0.47	88.33±1.24
Iso9	15.00±0.00	34.67±2.87
Iso10	15.67±0.47	57.33±1.70
Iso11	16.00±0.00	58.00±0.81
Iso12	13.00±0.82	52.33±4.02

Supporting Table 1. Inhibition zone and hydrophobicity of 12 isolates from *Macrobrachium rosenbergii*.

Organism	Max score	Total score	Query Coverage	E- value	Identity	Accession
P. pentosaceus wikim20	3.532e+05	3.033e+06	86%	0.0	99%	NZ_CP015918.1
P. pentosaceus SRCM100194	3.531e+05	3.027e+06	86%	0.0	99%	NZ_CP021927.1
P. pentosaceus ATCC 25745	3.422e+05	3.033e+06	87%	0.0	99%	NC_008525.1
P. pentosaceus SL4	2.864e+05	2.977e+06	85%	0.0	99%	NC_022780.1
P. pentosaceus SRCM100892	2.691e+05	2.968e+06	85%	0.0	99%	NZ_CP021474.1

Supporting Table 2. Top 5 closest genome neighborhoods of *P. pentosaceus* MR001 obtained from the NCBI Microbial Nucleotide BLAST.

No.	Gene	Annotation
1	arnC_1	Undecaprenyl-phosphate 4-deoxy-4-formamido-L-arabinose transferase
2	arnC_3	Undecaprenyl-phosphate 4-deoxy-4-formamido-L-arabinose transferase
3	cadA	Cadmium-transporting ATPase
4	clpP_2	ATP-dependent Clp protease proteolytic subunit
5	clpP_3	ATP-dependent Clp protease proteolytic subunit
6	copZ	Copper chaperone CopZ
7	cysL	HTH-type transcriptional regulator CysL
8	dnaN_2	Beta sliding clamp
9	drrA	Daunorubicin/doxorubicin resistance ATP-binding protein DrrA
10	drrB	Daunorubicin/doxorubicin resistance ABC transporter permease protein DrrB
11	fmnP	Riboflavin transporter FmnP
12	fruA_1	PTS system fructose-specific EIIABC component
13	fruA_2	PTS system fructose-specific EIIABC component
14	fruA_3	PTS system fructose-specific EIIB'BC component
15	gmuC_2	PTS system oligo-beta-mannoside-specific EIIC component
16	ssbA_2	Single-stranded DNA-binding protein A
17	IS family	IS6 family transposase IS1216E
18	maa_1	Maltose O-acetyltransferase
19	wcaJ	UDP-glucose:undecaprenyl-phosphate glucose-1-phosphate transferase
20	wbbI	Beta-1,6-galactofuranosyltransferase WbbI
21	IS family	IS1182 family transposase ISLac1
22	smc_1	Chromosome partition protein Smc
23	hin	DNA-invertase hin
24	malY	Protein MalY
25	mdtD_2	Putative multidrug resistance protein MdtD
26	mscL_2	Large-conductance mechanosensitive channel
27	mtaD	5-methylthioadenosine/S-adenosylhomocysteine deaminase
28	rhaR_1	HTH-type transcriptional activator RhaR
29	ribZ_2	Riboflavin transporter RibZ
30	smc_3	Chromosome partition protein Smc
31	ssbA_3	Single-stranded DNA-binding protein A
32	tarF_1	Teichoic acid poly(glycerol phosphate) polymerase
33	xerC_4	Tyrosine recombinase XerC

Supporting Table 3. Genes and their encoded proteins in *P. pentosaceus* MR001 were not detected or had sequence similarities of less than 50% in with the three known strains, *P. pentosa*ceus ATCC25745, SRCM100194 and wikim20.

Primer	Sequence (5′-3′)	Reference
ProPO-F	GAGATCGCAAGGGAGAACTG	Sánchez-Ortiz et al.[36]
ProPO-R	CGTCAGTGAAGTCGAGACCA	Sánchez-Ortiz et al.[36]
TGase-F	CCTCAGGATCTCCTTCACCA	Sánchez-Ortiz et al.[36]
TGase-R	TTGGGAAAACCTTCATTTCG	Sánchez-Ortiz et al.[36]
LvToll-F	ATGTGCGTGCGGATACATTA	Sánchez-Ortiz et al.[36]
LvToll-R	GGGTGTTGGATGTCGAGAGT	Sánchez-Ortiz et al.[36]
EF-F	GAACTGCTGACCAAGATCGACAGG	In this study
EF-R	GAGCATACTGTTGGAAGGTCTCCA	In this study
27f	AGAGTTTGATCCTGGCTCAG	Universal primer
1492r	GGTTACCTTGTTACGACTT	Universal primer
EntA-F	CCATGGAAGCCAGCCATCTTCT	In this study
EntA-R	GGTAAGACGCCAACGCCAAG	In this study
SrtA-F	ATGAGTAAAAAACTAAAAAATTGGTTAG	In this study
SrtA-R	TTAACGATTAAACTTTTCTGAAAACTC	In this study

Supporting Table 4. Primers used in this study.

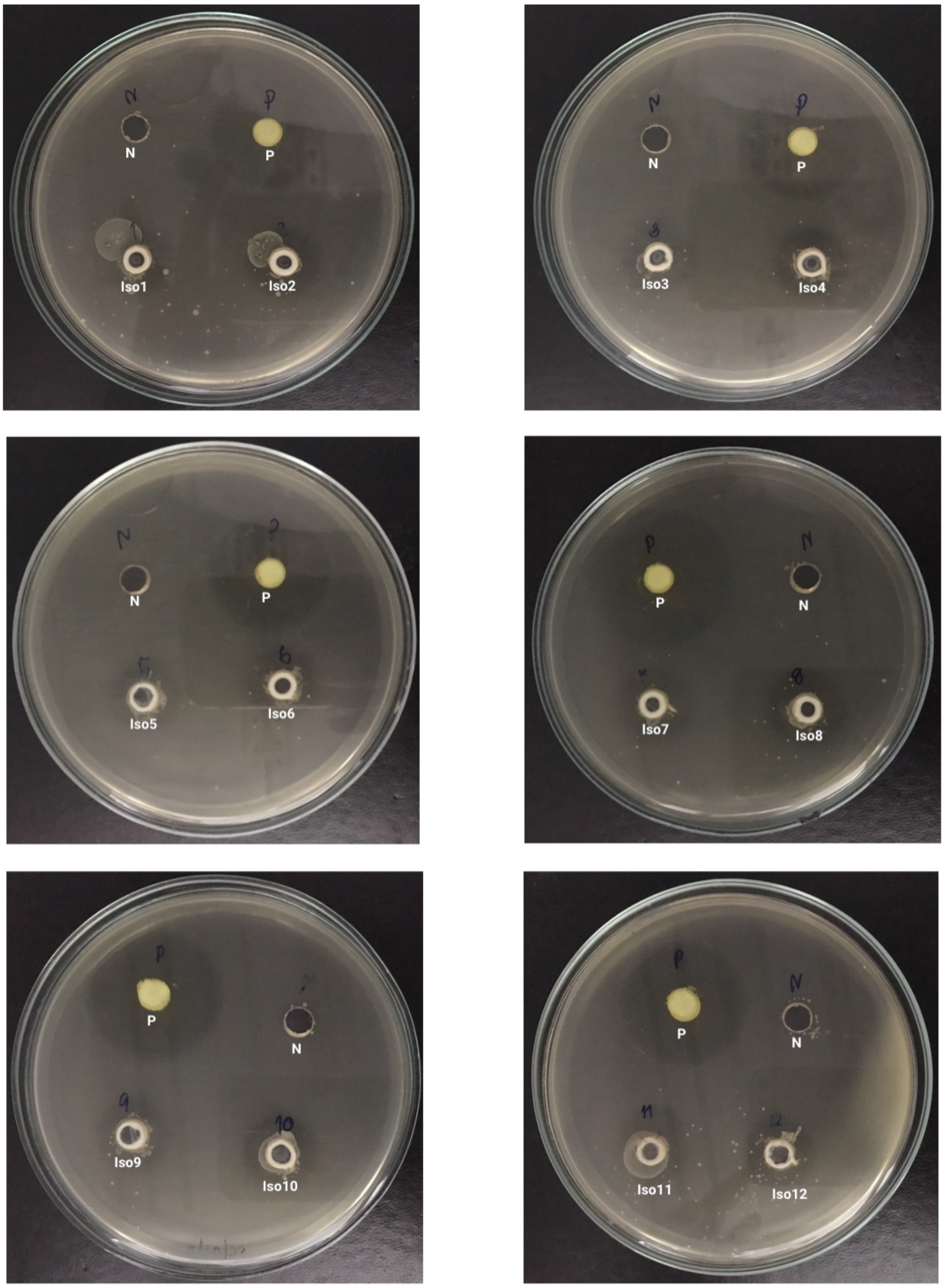

Supporting Figure 1. The antimicrobial activity against *V. harveyi* of 12 isolates. P is positive control (100 µg/ml of tetracycline); N is negative control (MRS broth).

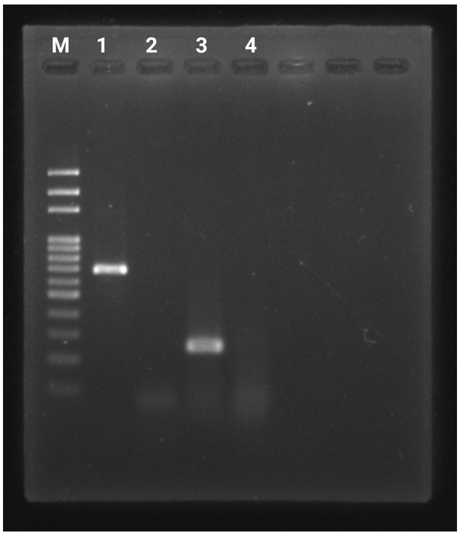

Supporting Figure 2. Transcripts of *sortase A* and *entrolysin A* from MR001 using RT-PCR. M: 100 bp DNA ladder, 1: PCR product of *sortase A*, 3: PCR product of *entrolysin A*, 2 and 4: negative control.
